# Mutational spectrum of acute myeloid leukemia patients with double *CEBPA* mutations based on next-generation sequencing and its prognostic significance

**DOI:** 10.18632/oncotarget.23873

**Published:** 2018-01-03

**Authors:** Long Su, YeHui Tan, Hai Lin, XiaoLiang Liu, Li Yu, YanPing Yang, ShanShan Liu, Ou Bai, Yan Yang, FengYan Jin, JingNan Sun, ChunShui Liu, QiuJu Liu, SuJun Gao, Wei Li

**Affiliations:** ^1^ Department of Hematology, The First Hospital, Jilin University, Changchun, China; ^2^ Department of Hematology, Chinese PLA General Hospital, Peking, China

**Keywords:** acute myeloid leukemia, *CEBPA* mutations, next generation sequencing, prognoses, Chinese population

## Abstract

The aim of this study was to profile the spectrum of genetic mutations in acute myeloid leukemia (AML) patients co-occurring with *CEBPA* double mutation (*CEBPA*^dm^). Between January 1, 2012, and June 30, 2017, 553 consecutive patients with *de novo* AML were screened for *CEBPA* mutations. Out of these, 81 patients classified as *CEBPA*^dm^ were analyzed further by a sensitive next-generation sequencing assay for mutations in 112 candidate genes. Within the *CEBPA* gene itself, we found 164 mutations. The most common mutated sites were c.936_937insGAG (n = 11/164, 6.71%) and c.939_940insAAG (n = 11/164, 6.71%), followed by c.68dupC (n = 10/164, 6.10%). The most common co-occurring mutations were found in the *CSF3R* (n = 16/81, 19.75%), *WT1* (n = 15/81, 18.52%), and *GATA2* (n = 13/81, 16.05%) genes. Patients with *CSF3R* mutations had an inferior four-year relapse-free survival (RFS) than those with the wild-type gene (15.3% versus 46.8%, respectively; *P* = 0.021). Patients with *WT1* mutations had an inferior five-year RFS compared with those without such mutations (0% versus 26.6%, respectively, *P* = 0.003). However, *GATA2*, *CSF3R*, *WT1* mutations had no significant influence on the overall survival. There were some differences in the location of mutational hotspots within the *CEBPA* gene, as well as hotspots of other co-occurring genetic mutations, between AML patients from Chinese and Caucasian populations. Some co-occurring mutations may be potential candidates for refining the prognoses of AML patients with *CEBPA*^dm^ in the Chinese population.

## INTRODUCTION

Mutations in the *CCAAT/enhancer binding protein α* (*CEBPA*) gene occur in 7%–15% of all acute myeloid leukemia (AML) cases. The subgroup of biallelic *CEBPA* mutations in AML patients has now been acknowledged in ‘The 2016 revision to the World Health Organization classification of myeloid neoplasms and acute leukemia’ as a definite entity, given its distinct biological and clinical features, as well as its prognostic significance [1]. *CEBPA* belongs to the basic-leucine zipper (b-ZIP) family of transcription factors whose C-terminal regions contain two highly conserved motifs: a DNA-binding motif rich in basic amino acids and a leucine zipper dimerization motif. They also contain two less conserved N-terminal transactivation domains (TADs) [2]. *CEBPA* mutations can occur across the whole gene, but cluster in two main hotspots: N-terminal frame-shift insertions/deletions—these cause translation of a 30 kDa protein from an internal ATG start site that lacks transactivation domain 1 and has a dominant negative effect over the full-length p42 protein; C-terminal mutations— these are generally in-frame insertions/deletions, in the DNA-binding or leucine zipper domains, that disrupt binding to DNA or dimerization [3].

AML patients with double *CEBPA* mutations (*CEBPA*^dm^) show a favorable outcome, which was also observed in our previous study [4]. Both others’ and our studies suggest that the frequency of *CEBPA* mutations (17.1%–21.6%) may be higher in Chinese patients with AML than what has been reported for populations of Western countries [4–5]. We also noticed some genetic differences between patients with AML from China and Western countries [4, 6–7]. Although the genetic profiling of AML patients with *CEBPA*^dm^ has been reported in previous studies [8–9], there is no data available for Chinese patients. Furthermore, the prognostic significance of co-occurring mutations remains unclear in patients with *CEBPA*^dm^. In this study, we screened 553 patients with *de novo* AML and profiled genetic mutations in those with *CEBPA*^dm^ (n = 81) by a sensitive next-generation sequencing assay. The prognostic significance of the top three co-occurring genetic mutations was also evaluated.

## RESULTS

### Patients’ characteristics

Of the 553 consecutive patients with *de novo* AML, *CEBPA* mutations were detected in 105 patients (18.99%), with 81 cases (14.65%) harboring double mutations and 24 cases (4.34%) harboring single mutations. Characteristics of the patients are summarized in Table 1. Most (60.49%) of the patients were morphological M1 and M2 subtypes according to the French–American–British (FAB) classification system. Of the 65 patients who underwent successful cytogenetic analysis, 60 cases (92.31%) presented with normal karyotypes.

**Table 1:** The characteristics of 81 AML patients with *CEBPA*^dm^

Characteristics	Number of patients	Percentage
Age (years), median (range)	44 (9 ~ 67)	
Gender		
Male	45	55.56%
Female	36	44.44%
FAB classification		
M1	2	2.47%
M2	47	58.02%
M4	24	29.63%
M5	5	6.17%
M6	3	3.70%
Cytogenetics		
Normal karyotype	60	92.31%
Abnormal karyotypes	5	7.69%
Peripheral blood		
White blood cells (×10^9/L)	17.10 [8.84, 62.64]	
Hemoglobin (g/L)	97.56 ± 27.85	
Platelets (×10^9/L)	23.00 [12.00, 41.00]	
Marrow blasts (%)	61.00 ± 17.38	

### *CEBPA* mutation screening based on next-generation sequencing

**Figure 1:**
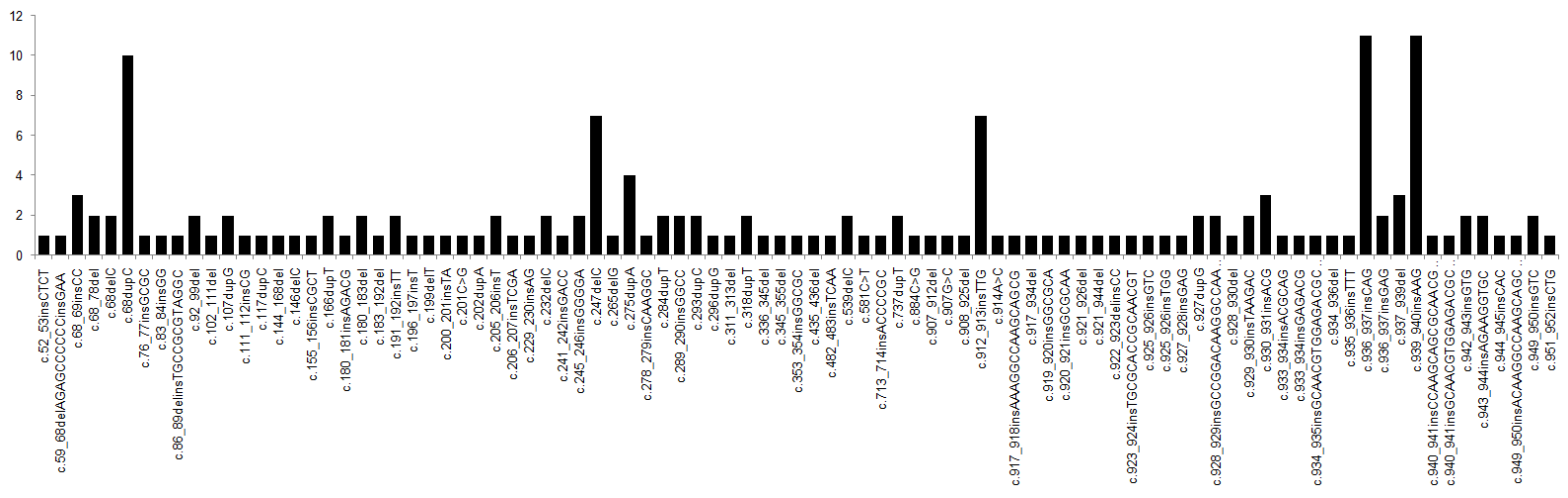
The mutation locations of *CEBPA* gene in AML patients with *CEBPA*^dm^

Among the 81 *CEBPA*^dm^ patients, 164 genetic mutations, classifiable into 91 different kinds, were detected in the *CEBPA* gene. The median mutation load was 45.3% (range: 4.5%–58.2%). The most common mutated sites were c.936_937insGAG (n = 11/164, 6.71%) and c.939_940insAAG (n = 11/164, 6.71%), followed by c.68dupC (n = 10/164, 6.10%), c.247delC (n = 7/164, 4.27%), c.275dupA (n = 7/164, 4.27%) (Figure 1). The majority of *CEBPA*^dm^ comprised frame-shift insertions or deletions (n = 83/164, 50.61%). The next most common were in-frame insertions or deletions (n = 72/164, 43.90%). The least common were missense mutations (n = 5/164, 3.05%), and stop-gain mutations (n = 4/164, 2.44%). A majority of *CEBPA*^dm^ patients (n = 64/81, 79.01%) showed a combination of an N-terminal frame-shift and a C-terminal in-frame mutation.

**Figure 2:**
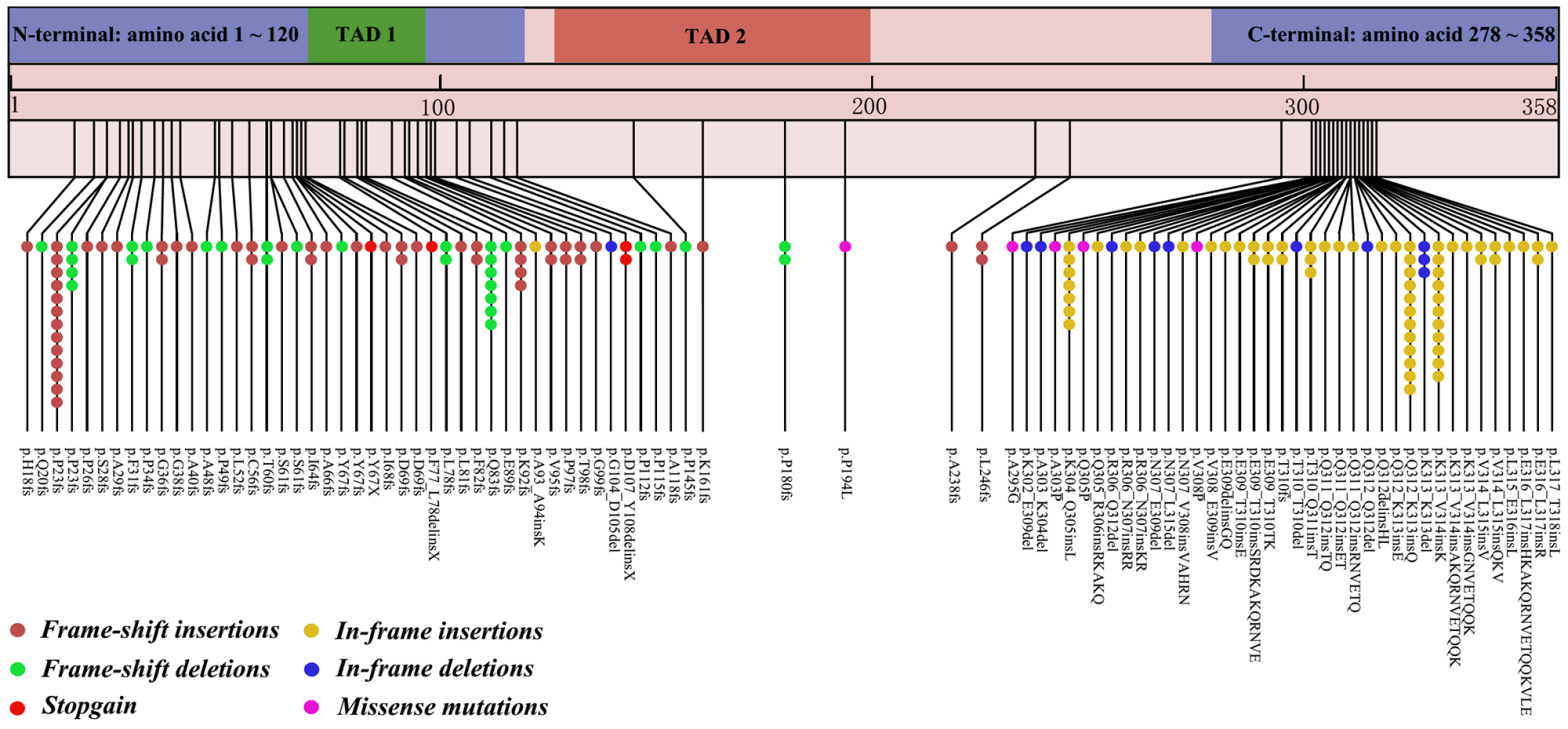
Amino acid alterations in CEBPA protein in AML patients with *CEBPA*^dm^

When the DNA sequences were translated into the corresponding amino acid sequences, the most common mutation site was p.Pro23fs (n = 17/164, 10.37%; 13 frame-shift insertions and four frame-shift deletions), followed by p.Gln312_Lys313insGln (n = 12/164, 7.32%), and p.Lys313_Val314insLys (n = 11/164, 6.71%; Figure 2).

### Correlation of the *CEBPA*^dm^ status to other molecular mutations

**Figure 3:**
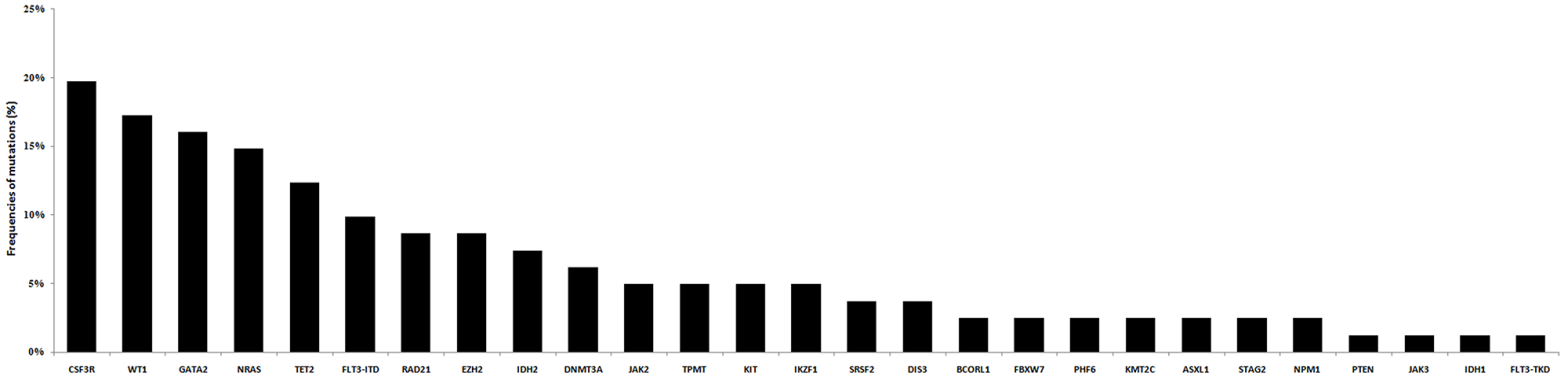
The distribution of co-occurring mutations in AML patients with *CEBPA*^dm^

Twenty-seven types of other molecular mutations were detected in patients with *CEBPA*^dm^. Seventeen patients (20.99%) had no additional molecular mutation, 23 (28.40%) had one, 20 (24.69%) had two, 12 (14.81%) had three, six had (7.41%) four, and three (3.70%) patients had five additional mutations (Supplementary Figure 1). *CSF3R* (n = 16), *WT1* (n = 15), and *GATA2* (n = 13), were the most common co-occurring mutations, with frequencies of 19.75%, 18.52%, and 16.05%, respectively (Figure 3).

Next, we analyzed the clinical characteristics of patients with mutations in other genes which co-occurred with frequencies exceeding 10%. These included mutations in the *CSF3R*, *WT1*, *GATA2*, *NRAS*, and *TET2* genes. *CSF3R* mutation was associated with a lower platelet (18.50 [11.25, 32.75] ×10^9^/L versus 23.00 [13.50, 47.00] ×10^9^/L; *u* = 2.873, *P* = 0.005) and higher leukocyte (53.57 [28.76, 73.39] ×10^9^/L versus 14.00 [7.33, 36.44] ×10^9^/L; *u* = 3.001, *P* = 0.030) counts as compared to the wild-type. *WT1* mutation was associated with a higher white blood cell count as compared to the wild-type (36.22 [13.22, 121.31] ×10^9^/L versus 16.36 [7.00, 50.00] ×10^9^/L; *u* = 2.024, *P* = 0.043). The average age of patients with a *WT1* mutation was less than the average age of those without one (28.14 ± 12.13 versus 42.56 ± 13.46; *t* = 3.702, *P* < 0.001; Table 2).

Table 2The characteristics of AML patients with different molecular mutations

**Table:** 

	*CSF3R*				*WT1*			*GATA2*		
	Mutations (n = 16)		Wide-type (n = 65)		Mutations (n = 14)	Wide-type (n = 67)		Mutations (n = 13)		Wide-type (n = 68)
Age (years)	38.88 ± 13.54		40.35 ± 14.52		28.14 ± 12.13	42.56 ± 13.46^*^		37.38 ± 14.75		40.57 ± 14.22
Gender										
Male	10 (62.50%)		35 (53.85%)		10 (71.43%)	35 (52.24%)		8 (61.54%)		37 (54.41%)
Female	6 (37.50%)		30 (46.15%)		4 (28.57%)	32 (47.76%)		5 (38.46%)		31 (45.59%)
FAB classification										
M1	0 (0.00%)		2 (3.08%)		1 (7.14%)	1 (1.49%)		0 (0.00%)		2 (2.94%)
M2	6 (37.50%)		33 (50.77%)		6 (42.86%)	33 (49.25%)		10 (76.92%)		29 (42.65%)
M4	9 (56.25%)		23 (35.38%)		6 (42.86%)	26 (38.81%)		2 (15.38%)		30 (44.12%)
M5	1 (6.25%)		4 (6.15%)		1 (7.14%)	4 (5.97%)		1 (7.69%)		4 (5.88%)
M6	0 (0.00%)		3 (4.62%)		0 (0.00%)	3 (4.48%)		0 (0.00%)		3 (4.41%)
Cytogenetics										
Normal karyotype	12 (92.31%)		48 (92.31%)		9 (100.00%)	51 (91.07%)		11 (100.00%)		49 (90.74%)
Abnormal karyotypes	1 (7.69%)		4 (7.69%)		0 (0.00%)	5 (8.93%)		0 (0.00%)		5 (9.26%)
Peripheral blood										
White blood cells (×10^9/L)	53.57 [28.76, 73.39]		14.00 [7.33, 36.44]^*^		36.22 [13.22, 121.31]	16.36 [7.00, 50.00] ^*^		16.45 [11.44, 67.81]		18.06 [8.48, 62.64]
Hemoglobin (g/L)	96.00 ± 25.10		97.94 ± 28.65		84.71 ± 27.94	100.24 ± 27.27		86.15 ± 28.57		99.74 ± 27.38
Platelets (×10^9/L)	18.50 [11.25, 32.75]		23.00 [13.50, 47.00]^*^		23.00 [10.75, 46.50]	23.00 [12.00, 40.00]		25.00 [9.50, 43.50]		23.00 [12.00, 39.75]
Marrow blasts (%)	53.63 ± 18.55		63.00 ± 16.66		62.07 ± 20.14	60.75± 16.86		59.23 ± 12.45		61.37 ± 18.31

**Table:** 

	*NRAS*		*TET2*	
	Mutations (n = 12)	Wide-type (n = 69)	Mutations (n = 10)	Wide-type (n = 71)
Age (years)	37.38 ± 14.75	40.57 ± 14.22	44.10 ± 11.20	39.49 ± 14.61
Gender				
Male	6 (50.00%)	39 (56.52%)	3 (30.00%)	42 (59.15%)
Female	6 (50.00%)	30 (43.48%)	7 (70.00%)	29 (40.85%)
FAB classification				
M1	1 (8.33%)	1 (1.45%)	1 (10.00%)	1 (1.41%)
M2	6 (50.00%)	33 (47.83%)	6 (60.00%)	33 (46.48%)
M4	2 (16.67%)	30 (43.38%)	3 (30.00%)	29 (40.85%)
M5	3 (25.00%)	2 (2.90%)	0 (0.00%)	5 (7.04%)
M6	0 (0.00%)	3 (4.35%)	0 (0.00%)	3 (4.23%)
Cytogenetics				
Normal karyotype	6 (75.00%)	54 (94.74%)	9 (90.00%)	51 (92.73%)
Abnormal karyotypes	2 (25.00%)	3 (5.26%)	1 (10.00%)	4 (7.27%)
Peripheral blood				
White blood cells (×10^9/L)	25.70 [9.72, 93.26]	17.10 [8.43, 62.64]	34.72 [21.35, 72.37]	16.36 [7.66, 50.00]
Hemoglobin (g/L)	86.15 ± 28.57	99.74 ± 27.38	87.80 ± 27.35	98.93 ± 27.83
Platelets (×10^9/L)	22.00 [14.00, 23.00]	23.00 [12.00, 42.00]	23.00 [12.50, 24.50]	23.00 [12.00, 45.00]
Marrow blasts (%)	59.23 ± 12.45	61.37 ± 18.31	69.49 ± 12.60	59.69 ± 17.72

^*^ Compared with patients with mutations, *P* < 0.05.

Co-occurring mutations were categorized as falling into various pathways and gene families: tyrosine kinase pathway, transcription factor gene, tumor suppressor gene, DNA methylation gene, chromatin-modifier gene, cohesion molecule gene, spliceosome complex gene, and others. The most frequent mutation involved genes affecting the tyrosine kinase pathway (33.33%), followed by DNA methylation (15.94%), and tumor suppressor (13.77%) gene families (Supplementary Figure 2).

### Treatment response and long-term outcome

**Figure 4:**
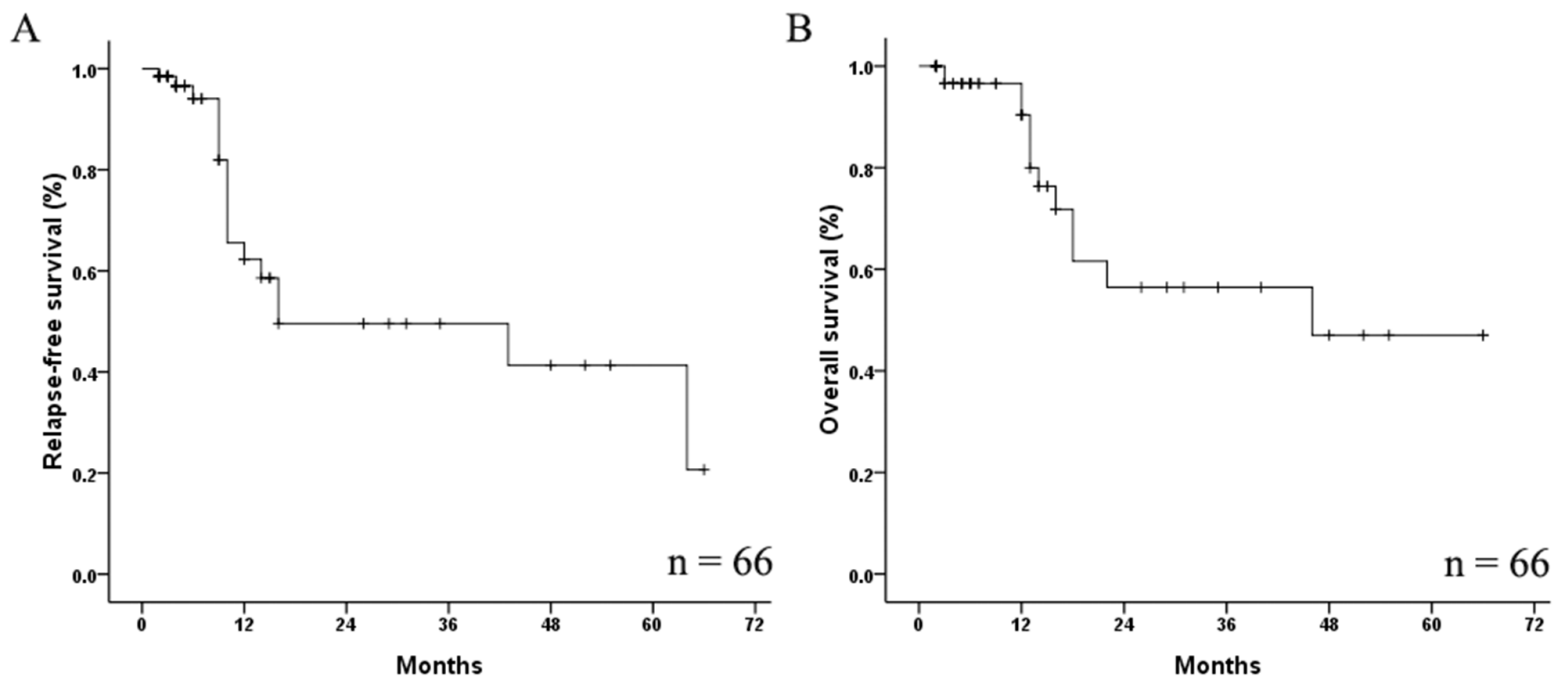
Relapse-free survival **(A)** and overall survival **(B)** in AML patients with *CEBPA*^dm^.

For 67 patients received induction therapy, 50 patients achieved complete remission (CR), 14 achieved partial remission (PR), and the remaining three cases were classified as non-remission (NR) after one course of chemotherapy. *CSF3R*, *WT1*, and *GATA2* mutations had no influence on the CR rate (*P* = 0.320, *P* = 0.130, and *P* = 0.158 respectively). Finally, 66 cases who achieved CR entered long-term follow-up. The follow-up time ranged from two to 66 months (median: 8 months). In total, 18 patients relapsed, and 13 patients died. Five-year relapse-free survival (RFS) (Figure 4A) and overall survival (OS) (Figure 4B) rates were 20.7% and 47.0%, respectively.

**Figure 5:**
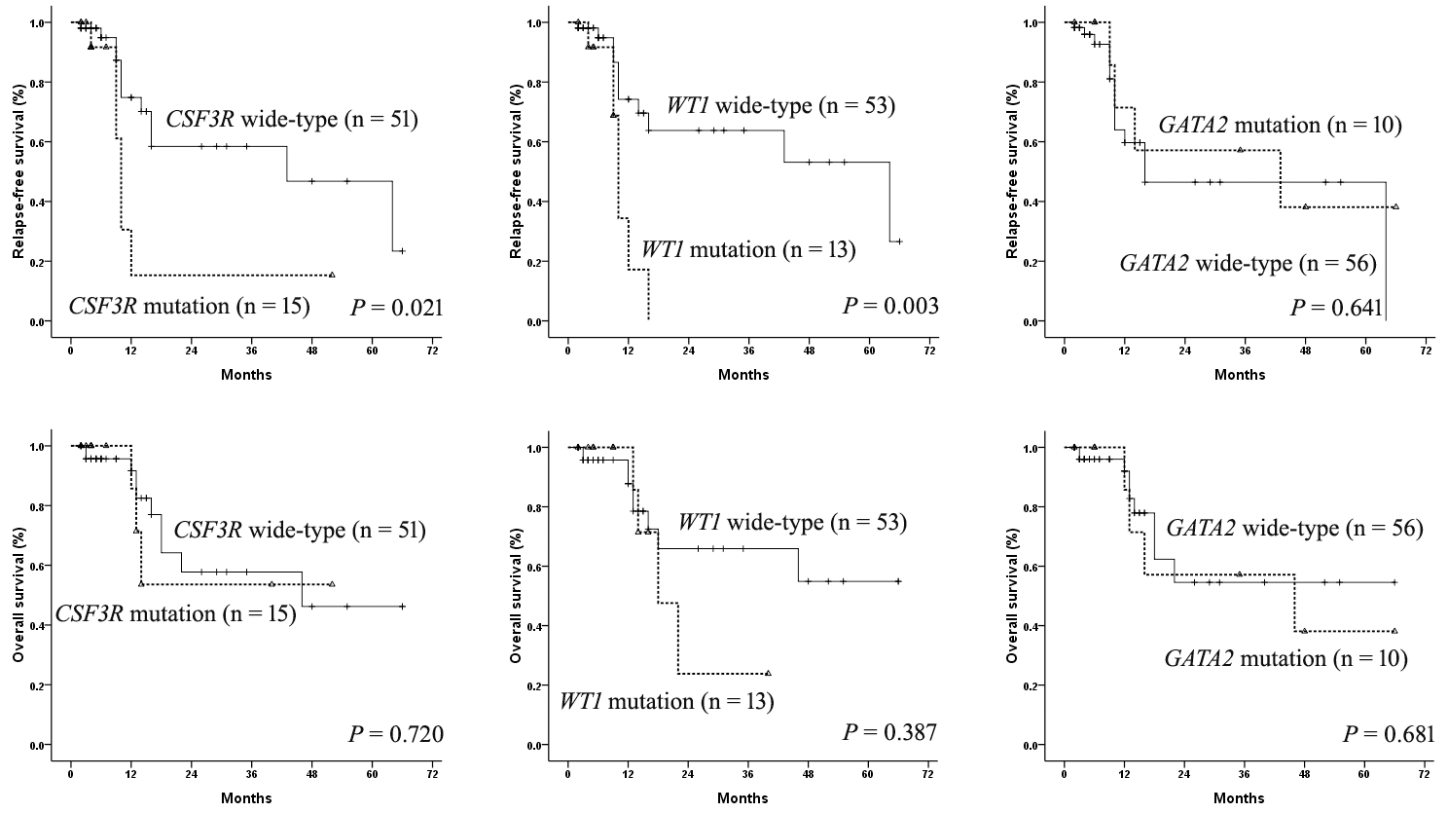
The influence of *CSF3R*, *WT1*, and *GATA2* mutations on outcomes in AML patients with *CEBPA*^dm^

We also evaluated the prognostic significance of *CSF3R*, *WT1*, *GATA2* mutations in patients with *CEBPA*^dm^. The four-year RFS of patients with *CSF3R* mutations was 15.3%, which was lower than those with wild-type *CSF3R* (46.8%) (*P* = 0.021). The median RFS of patients with mutated and wild-type *CSF3R* were 10 and 43 months, respectively. Patients with *WT1* mutations had an inferior five-year RFS compared with those without the mutations (0% versus 26.6%, *P* = 0.003). The median RFS of patients with mutated and wild-type *WT1* were 10 and 64 months, respectively. The five-year RFS rates were 38.1% and 46.4% in patients with the mutated and wild-type *GATA2*, respectively (*P* = 0.641). *GATA2*, *CSF3R*, *WT1* mutations had no significant influence on OS in this study (Figure 5).

## DISCUSSION

AML is a heterogeneous disease, and DNA sequencing can offer clues to its etiology and predict prognoses of patients with AML. With the development of new sequencing technology, more and more genetic mutations are being identified in AML patients [10]. In our previous studies, we observed some differences in genetic alterations between AML patients from China and Western countries [4, 6–7]. The frequencies of *NPM1* (15.4%) and *FLT3*-ITD mutations (14%) are lower in AML patients from China [7], whereas the frequency of *CEBPA* mutations is higher (17.1%) [4]. These results accord with the literature published by others from China [5, 11-12]. It is known that AML with *CEBPA*^dm^ indicates a favorable outcome, which was also confirmed in our cohort of patients [4]. However, it is unknown whether there are genetic differences among the geographic or ethnic subgroups of AML patients with *CEBPA*^dm^.

The present subset of AML patients was derived from 553 consecutive patients with *de novo* diagnoses, which avoided selection bias. The majority (60.49%) of patients presented with M1 and M2 subtypes, according to the FAB classification system. A normal karyotype was present in 92.31% of the patients, while aberrant karyotypes included del(9q) and +8 trisomy. Only two patients with *NPM1* mutation were detected in this study. All these features are consistent with previous reports [3, 8].

We found that the most common *CEBPA* mutation types were frame-shift insertions or deletions, followed by in-frame insertions or deletions, which is in accord with previous studies [3, 8]. A combination of an N-terminal frame-shift and a C-terminal in-frame mutation was present in the majority of patients in this study, which was also reported previously [8–9]. Fasen *et al*. reported that the most frequent mutation site was p.Lys313del, followed by p.His24Alafs, and p.Gln312del [8]. However, we observed a different result. The most common mutation site in the present study was p.Pro23fs, followed by p.Gln312_Lys313insGln, and p.Lys313_Val314insLys. We profiled for genetic mutations co-occurring in *CEBPA*^dm^ AML patients. Interestingly, we observed that the percentage of patients with three or more co-occurring molecular mutations was higher in this study than in previous studies (25.93% versus 2.88%, respectively, *χ*^2^ = 21.412, *P* < 0.001; [8]). We hypothesize that these differences between AML patients from Chinese and Caucasian populations may be due to their differing ethnic backgrounds.

Among AML patients with *CEBPA*^dm^, Grossmann *et al.* from Germany reported that *TET2* was found to be the most frequently mutated gene (34.0%), followed by *GATA2* (21.0%), and *WT1* (13.7%) genes [9]. Another German group reported that the top-three mutated genes were *TET2* (15.7%), *ASXL1* (13.7%), and *WT1* (13.6%) [8]. The top-three mutated genes identified in this study were *CSF3R* (19.75%), *WT1*(18.52%), and *GATA2* (16.05%).

The frequency of *GATA2* mutations in *CEBPA*^dm^ patients in this study (16.05%) was lower than that reported in previous studies [9, 13]. There are still some controversies regarding the prognostic significance of *GATA2* mutations in patients with *CEBPA*^dm^ [9, 13-15]. Grossmann*et al*. reported that *GATA2*-mutated patients show a longer OS than *GATA2* wild-type cases (n = 95; [9]). Hou and colleagues observed that among patients with *CEBPA*^dm^, those with *GATA2* mutations had a trend of better OS and RFS than those without (n = 62; [13]). In univariate analysis, *GATA2* mutations were associated with better event-free survival (EFS) and OS (*P* = 0.03 and *P* = 0.041, respectively; n = 98; [14]). However, no significant difference in CR rate, RFS, and OS was also observed in *CEBPA*^dm^ patients with and without *GATA2* mutations (n = 113; [15]). In the present study, we found that *GATA2* mutations had no influence on CR, RFS and OS. Due to the relatively small number of patients in these studies, further research is still needed to evaluate the prognostic significance of *GATA2* mutation in patients with *CEBPA*^dm^. Furthermore, we argue that ethnicity should also be taken into account when conducting analyses.

Recently, Lavallée *et al.* from Canada reported that *CSF3R* mutations were the most frequent mutations (29%) in AML patients with *CEBPA*^dm^ [16]. Maxson and colleagues confirmed those findings in a cohort of pediatric patients with AML. They found a significant enrichment of *CSF3R* mutations (46%) among the *CEBPA*-mutated AML patients in America [17]. A high frequency of *CSF3R* mutations was also observed in our cohort of AML patients. In accordance with a previous study (n = 11/19, 57.89%) [17], we also found the majority of *CSF3R* mutations (n = 11/16, 68.75%) were p.T618I. Collectively, these findings suggest that *CEBPA*^dm^ AML patients may benefit from treatment with Janus kinase inhibitors.

Although AML with *CEBPA*^dm^ indicates a favorable outcome, recent data show that more than 50% of the patients finally relapsed when consolidated with chemotherapy alone [18]. Hence, a new marker is needed to stratify patients with *CEBPA*^dm^. Patients with *CSF3R* and *WT1* mutations showed inferior RFS compared with those with the wild-type genes. As a result, *WT1* and *CSF3R* mutations may be adopted as potential markers to stratify patients with *CEBPA*^dm^ in the Chinese population.

Consistent with a previous study [19], we also found that the most frequent mutations in patients with *CEBPA*^dm^ occurred in the tyrosine kinase signaling pathway. Exploration or evaluation of drugs targeting these pathways, and translational research integrating these molecular findings, may improve the treatment of patients with *CEBPA*^dm^.

In summary, we found that there were some differences in hotspots of *CEBPA* mutations, and in hotspots of co-occurring genetic mutations, between AML patients from Chinese and Caucasian populations. Some of the co-occurring mutations may even be potential candidates, for treating patients with *CEBPA*^dm^, specific to the Chinese population. The continuation of such studies may uncover more mutational differences based on ethnicity, which may similarly reveal information pertinent to research into the etiology of AML and treatment of AML patients with *CEBPA*^dm^.

## MATERIALS AND METHODS

### Patients and treatment

From January 1, 2012, to June 30, 2017, 553 consecutive patients with *de novo* AML were screened for *CEBPA* mutations from our center and Chinese People's Liberation Army (PLA) General Hospital. They were categorized into FAB subtypes (M0–M7) based on morphological diagnoses [20] (Supplementary S3). Patients in this study were treated with the standard ‘3+7’ regimen (darubicin/idarubicin + cytarabine) or CAG (aclarubicin + cytarabine + G-CSF) regimen (for some elderly patients) for induction therapy. The response was assessed by bone marrow aspiration performed on days 14 and 28. The first consolidation therapy was the same as that generally used to achieve CR. Three to four courses of scheduled, high-dose cytarabine, at 2–3 g/m^2^, were administrated for consolidation therapy. Five patients with *CEBPA*^dm^ received allo-HSCT. All of the participating patients gave informed consent prior to enrolment in the study. This study was approved by the ethics committee of Jilin University and Chinese PLA General Hospital, and conducted in accordance with the Declaration of Helsinki.

### Cytogenetic analysis

Standard culturing and chromosome-banding techniques were used to analyze the karyotypes. Their clonal abnormalities were defined and described according to the International System for Human Cytogenetic Nomenclature [21].

### Molecular mutations screening by next-generation sequencing

Eighty-one patients with *CEBPA*^dm^ were analyzed by a sensitive next-generation sequencing (NGS) assay for 112 genes (see Table 3). The NGS assay was performed as previously described [22], covering 654 coding regions, and approximately 2610000 base pairs. A NimbleGen SeqCap EZ Choice kit was used according to the manufacturer's protocol with some modifications. Multiplexed libraries were sequenced using 75-bp paired-end runs on an Illumina NextSeq 550AR system. Reads were aligned using the Burrows-Wheeler alignment (BWA) tool (version 0.7.5a) against human genomic reference sequences (HG19, NCBI build 37). To identify single nucleotide polymorphisms (SNPs) and short insertions and deletions (INDELs), MuTect2 operation was performed with recommended parameters. All mutations were annotated by the ANNOVAR software. A subset of somatic mutations was randomly selected for validation using Sanger sequencing. Cell line dilutions were prepared for evaluation of sensitivity and specificity. For AML patients in this study, the SCARF file was converted to the FASTQ format by the CASAVA software (version 1.8, Illumina). Raw sequence reads were filtered with an indigenous program. Reads with more than 5% N bases or in which at least 50% bases had Q ≤5 were eliminated. The remaining reads were aligned using the BWA tool to the human genomic reference sequences (HG19, NCBI build 37) with certain parameters (mem -t 10 -k 32 -M). To decrease PCR duplication bias, the resulting Bam files were processed with Sam tools. Only unique reads were delivered for analyses. For identification of SNP and indel, MuTect2 operation was performed with recommended parameters. All mutations were annotated by the ANNOVAR software using the following resources: all annotated transcripts in RefSeq Gene; known constitutional polymorphisms as reported in human variation databases, such as 1000 Genomes (release date 20130308), the Exome Aggregation Consortium (ExAC release date 20151129) and dbSNP (version 135) were download from ANNOVAR; known somatic variations in myeloid and other malignancies as reported in COSMIC (version 70). To identify high-confidence somatic variants in AML samples in the absence of matched control samples, the following criteria were used: removal of all variants within intronic, UTR and intergenic regions, and retention of only nonsynonymous, frame-shift and stop-gain mutations in exonic regions; removal of all variants present in at least one of 81 healthy individuals; removal of all variants with one of the following features in MuTect results: mutation depth of less than four, Phred-scaled *p*-value using Fisher's exact test to detect strand bias of more than 60, mapping quality lower than 40. Because we lacked matched normal samples, somatic mutations could not be selected by comparing a tumor with a matched, normal sample. Thus, a series of steps were used to remove germline mutations and harmless mutations. Mutations were removed unless they satisfied all of the following conditions: the mutation depth was more than four; the mutation occurred in an exonic region; the mutation function was not “synonymous SNV”; the annotation from ClinVar was not “benign” or the mutation did not appear in a dbSNP135 or the 1000 Genomes Project (2012 Feb) database.

**Table 3:** Mutations of 112 genes analyzed in this study

Number	Gene	Number	Gene	Number	Gene	Number	Gene
1	*CEBPA*	29	*CALR*	57	*CCND1*	85	*KMT3A*
2	*NPM1*	30	*CSF3R*	58	*CD79B*	86	*MAP2K4*
3	*FLT3-ITD*	31	*SH2B3*	59	*CDA*	87	*MAP3K7*
4	*FLT3-TKD*	32	*IKZF1*	60	*CREBBP*	88	*MDM2*
5	*KIT*	33	*ABL*	61	*CRLF2*	89	*MEF2B*
6	*DNMT3A*	34	*NOTCH1*	62	*CSF1R*	90	*MLH1*
7	*IDH1*	35	*FBXW7*	63	*CTLA4*	91	*MTHFR*
8	*IDH2*	36	*TPMT*	64	*CUX1*	92	*NF2*
9	*MLL*	37	*CDKN2A*	65	*CYP2C19*	93	*NOTCH2*
10	*TET2*	38	*ATM*	66	*CYP3A4*	94	*NQO1*
11	*WT1*	39	*HRAS*	67	*DIS3*	95	*NT5C2*
12	*RUNX1*	40	*RB1*	68	*DNAH9*	96	*NTRK1*
13	*KRAS*	41	*MYD88*	69	*E2A*	97	*NTRK2*
14	*NRAS*	42	*ABCB1*	70	*EGFR*	98	*PDGFRA*
15	*ASXL1*	43	*ABCC3*	71	*ERCC1*	99	*PIGA*
16	*PHF6*	44	*AKT2*	72	*ERG*	100	*PIK3CA*
17	*TP53*	45	*AKT3*	73	*FAM46C*	101	*PTEN*
18	*SF3B1*	46	*AMER1*	74	*GATA1*	102	*RAD21*
19	*SRSF2*	47	*APC*	75	*GATA2*	103	*SMAD4*
20	*U2AF1*	48	*ATRX*	76	*GNAS*	104	*SMC1A*
21	*ZRSR2*	49	*BCL2*	77	*GSTM1*	105	*SMC3*
22	*EZH2*	50	*BCOR*	78	*GSTP1*	106	*STAG2*
23	*CBL*	51	*BCORL1*	79	*ID3*	107	*STAT5A*
24	*JAK2*	52	*BRAF*	80	*IL17R*	108	*STAT5B*
25	*SETBP1*	53	*CACNA1E*	81	*JAK1*	109	*SYK*
26	*ETV6*	54	*CARD11*	82	*JAK3*	110	*TERC*
27	*PTPN11*	55	*CBLB*	83	*KDM6A*	111	*TRAF3*
28	*MPL*	56	*CBLC*	84	*KMT2C*	112	*XRCC1*

### Statistics

Statistics Package for Social Sciences (SPSS) software (Version 17.0, SPSS Inc., Chicago, IL, USA) was used to calculate the statistical difference. For categorical variables, the *Chi-square* test or *Fisher's exact* test was used to assess the statistical significance of differences between groups. Independent-samples *t*-test or Mann-Whitney *U*-test was used to compare differences between groups for continuous variables. Kaplan-Meier method was employed for survival analysis, and the log-rank test was used to compare differences between groups. *P* < 0.05 was considered significant in all tests.

## SUPPLEMENTARY MATERIALS AND FIGURES


