# Supplementary material for: Mutational spectrum of acute myeloid leukemia patients with double CEBPA mutations based on next-generation sequencing and its prognostic significance
Source: Oncotarget. 2018 Jan 3;9(38):24970–9. doi: 10.18632/oncotarget.23873 (PMC5982761; doi:10.18632/oncotarget.23873)
Supplement: Supplementary file 1 [file oncotarget-09-24970-s001.pdf]

Mutational spectrum of acute myeloid leukemia patients with double *CEBPA* mutations based on next-generation sequencing and its prognostic significance

SUPPLEMENTARY MATERIALS

A brief describe of FAB and WHO classification

French–American–British (FAB) classification system of AML was based on morphological diagnosis of blasts in bone marrow, by which patients can be divided into M0 ~ M7. This classification criteria was first published in *British Journal of Haematology* in 1976. World Health Organization (WHO) classification of AML was mainly based on molecular or cytogenetic analysis, combining with morphology and immunophynotype. The 2016 revision to the World Health Organization classification of myeloid neoplasms and acute leukemia was published in *Blood* recently. Furthermore, FAB subtypes are foundations for both FAB and WHO classifications.

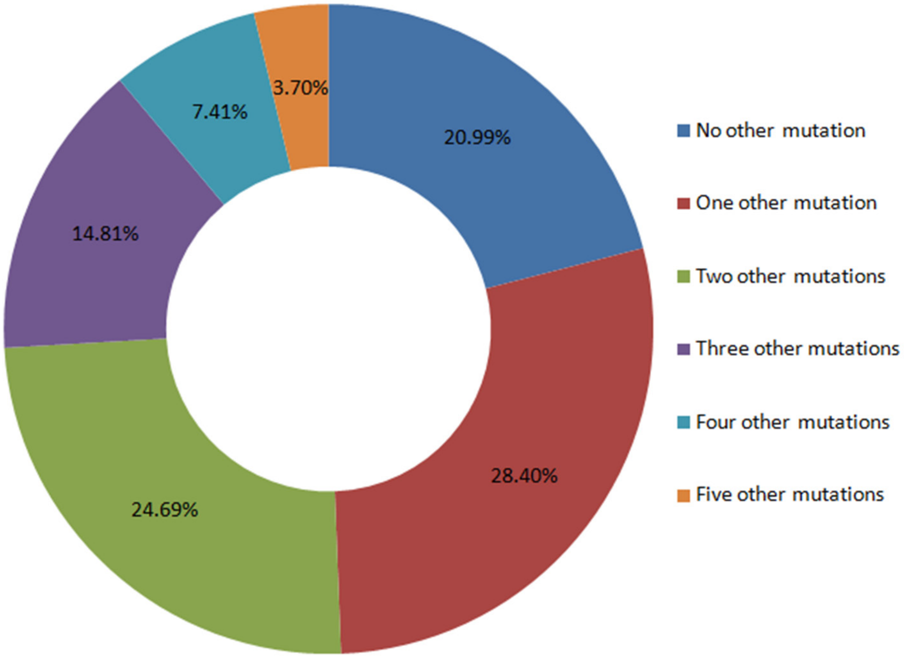

Supplementary Figure 1: Frequencies of co-occurring mutations in AML patients with *CEBPA*^{dm}.

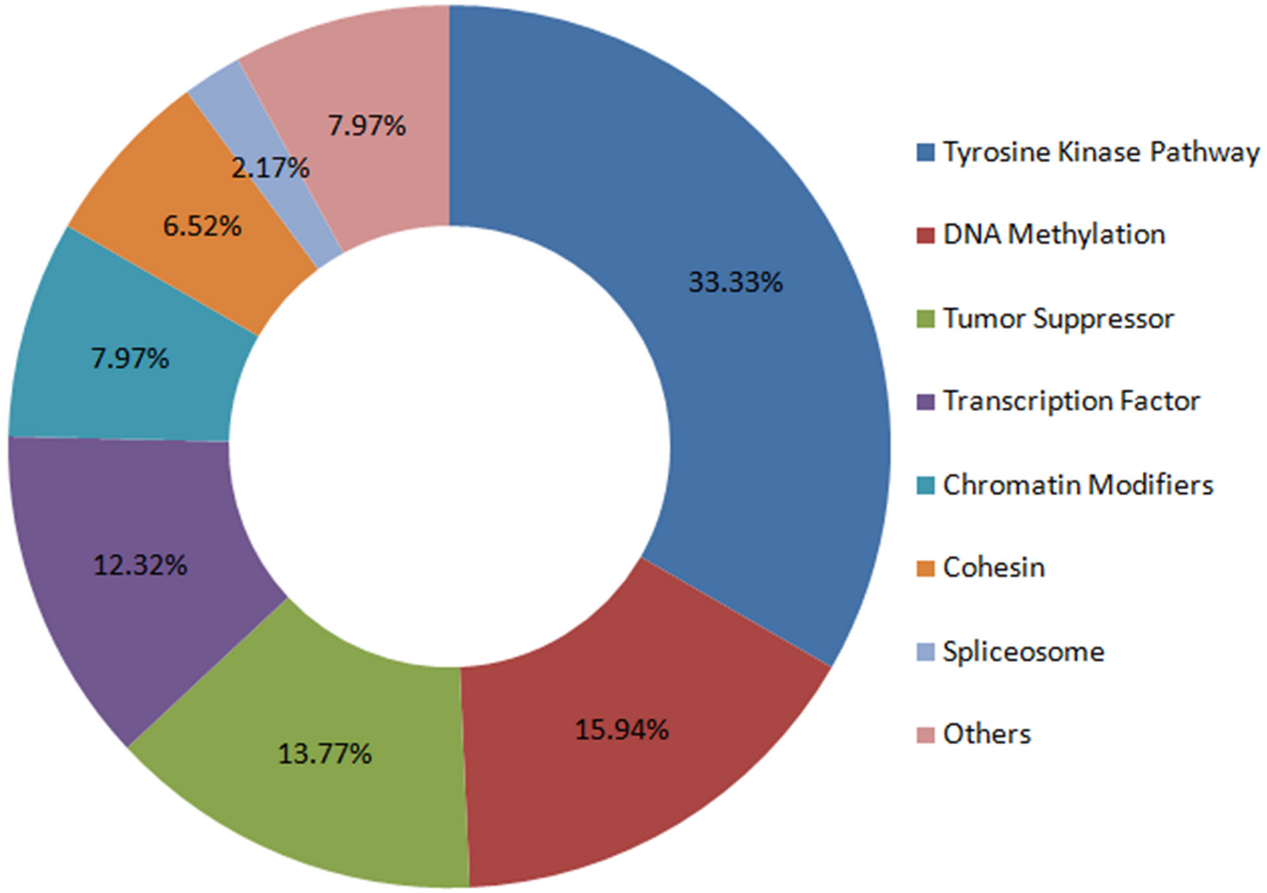

Supplementary Figure 2: Classification of co-occurring mutations based on biological functions.
